# Anomalous origin of the right coronary artery from the pulmonary artery with a bicuspid aortic valve in a 57-year-old patient

**DOI:** 10.1016/j.xjtc.2026.102297

**Published:** 2026-03-02

**Authors:** Raisa Bushra, Hugh Jacobs, Kaitlyn Gilbert, Gianni Angelini, Eltayeb Mohamed Ahmed

**Affiliations:** a Cardiac Surgery Department, Bristol Heart Institute, Bristol, United Kingdom; b Bristol Medical School, University of Bristol, Bristol, United Kingdom

**Figure:**
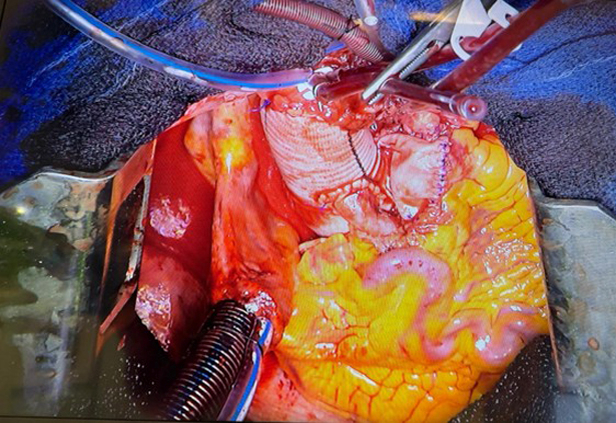
Anomalous origin of the right coronary artery from the pulmonary artery.

**Central Message**

ARCAPA with bicuspid aortic valve in adults is rare; early recognition and surgical correction prevent myocardial ischemia and improve outcomes.

The anomalous origin of the right coronary artery from the pulmonary artery (ARCAPA) is an exceedingly rare congenital anomaly, documented in fewer than 0.002% of all cases of congenital heart defects.^1^ Anomalous origin of the left coronary artery from the PA is more common in adults than ARCAPA.^2^ The unique pathophysiology of ARCAPA cause retrograde blood flow from the PA, if left untreated at birth or early childhood, can cause severe symptoms like myocardial ischemia, heart failure, or sudden cardiac death. However, patients with ARCAPA with good collateral blood supply to the territory are often asymptomatic or present with nonspecific symptoms, making diagnosis challenging.^3^ Isolated ARCAPA is common, but it can be associated (25-30%) with other structural congenital heart diseases like a ventricular septal defect, tetralogy of Fallot, aortopulmonary window, atrial septal defects, and aortic stenosis.^1^ This case report highlights ARCAPA with a bicuspid aortic valve in a 57-year-old patient to emphasize the atypical clinical presentation, diagnostic approach, and management strategies.

## Clinical Presentation

**Figure 1:**
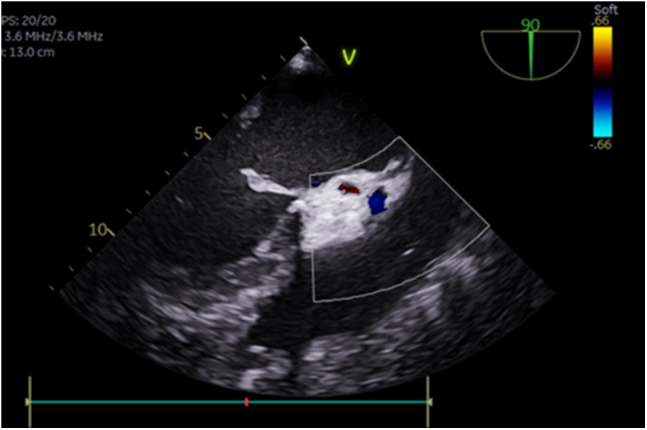
Echocardiogram showing anomalous origin of the right coronary artery.

**Figure 2:**
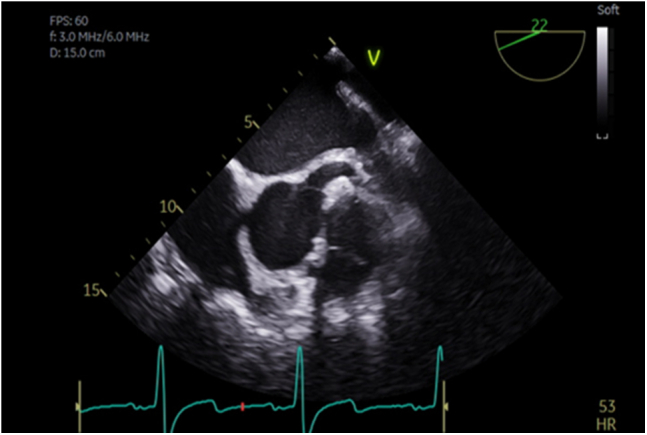
Echocardiogram picture of right coronary artery origin and course.

A 57-year-old man with a history of epilepsy and an extensive smoking history presented with progressively worsening shortness of breath (New York Heart Association functional class II-III) without any chest pain (Canadian Cardiovascular Society class 0). Initially, transthoracic echocardiogram (TTE) revealed severe bicuspid aortic valve stenosis with reduced left ventricular systolic function, as indicated by an ejection fraction of 31% (Figures 1 and 2). For preoperative workup, a computed tomography (CT) scan of the aorta was performed, which also showed severe thickening and calcification of the aortic valve with fusion of the right coronary cusp and left coronary cusp and a dilated aortic root that measured 45 × 38 mm; the midascending aorta measured 48 × 48 mm.

To further evaluate his condition for potential surgical intervention, a CT coronary angiogram was performed to exclude coronary artery disease. However, it revealed a rare congenital anomaly: ARCAPA. Imaging confirmed that the RCA originated from the PA and was collateralized by the left anterior descending artery (Video 1), creating a left-to-right shunt and severe bicuspid aortic stenosis, which was considered the cause of his symptoms.

## Management

The patient was discussed by the aortic multidisciplinary team, and the outcome was an aortic root replacement with mechanical aortic valve replacement and ARCAPA correction.

**Figure 3:**
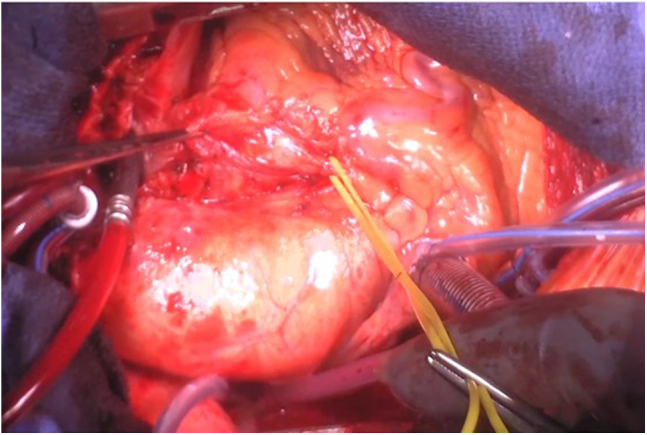
Right coronary artery originating from the pulmonary artery before correction.

**Figure 4:**
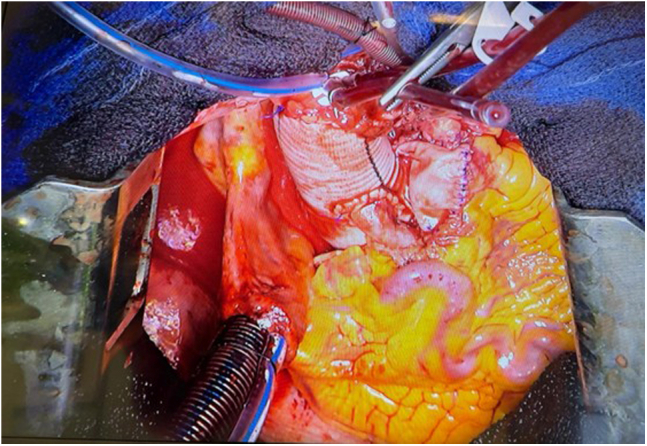
Surgical correction of right coronary artery and aortic root showing right coronary button repositioned to the aorta and patch repair of the pulmonary artery.

After standard cardiac surgery preparation with sternotomy and cannulation and initiation of the cardiopulmonary bypass machine, intraoperative findings confirmed the anomalous origin of the RCA from the PA in the aortopulmonary artery groove with a severely calcified bicuspid aortic valve. Initial myocardial protections were done using cold blood antegrade cardioplegia while occluding the RCA to avoid a left-to-right shunt (Figure 3). The ARCAPA correction was done by opening the PA, careful dissection around the RCA, and preparing the right coronary button for reimplantation to the ascending aorta. The PA was closed with a heterogeneous pericardial patch. Mechanical aortic valve replacement with aortic root replacement was performed with an interposition tube graft, and the right and left coronary buttons were reimplanted to the graft to restore the normal coronary anatomy (Figure 4) (Video 2).

The procedure was uneventful, and the patient was extubated on the first postoperative day. His postoperative echocardiogram showed satisfactory surgical outcome. He was discharged on postoperative day 11 with lifelong anticoagulation initiated. We performed a postoperative follow-up echocardiogram at 6 months to evaluate his cardiac function. His left ventricular function significantly improved from 31% to 52% and his right ventricle function remained unchanged with normal right ventricle size.

## Discussion

ARCAPA is a rare congenital anomaly that frequently remains undiagnosed due to its asymptomatic or nonspecific presentation.^3^ In this condition, the RCA arises from the PA, creating a left-to-right shunt from the higher-pressure left coronary artery to the lower-pressure RCA, eventually to the PA.^1^ This shunt can lead to a coronary steal phenomenon, resulting in myocardial ischemia, arrhythmias, or even sudden cardiac death while remaining undiagnosed.^1^^,^^2^

The diagnosis of ARCAPA presents a considerable challenge, especially when the patient is asymptomatic or has atypical symptoms.^3^ With the advancement of modern technology, multimodality imaging, including TTE, CT coronary angiography, and cardiac magnetic resonance imaging, is making diagnosis more accurate. These investigation tools are not only essential for correct diagnosis but also for planning appropriate surgical interventions.^3^ TTE, a first-line investigation, gives information about valve conditions or ventricle dysfunction. On the other hand, CT coronary angiography provides detailed anatomical information about the anomalous vessel, including its origin, branches, calcification, ventricle function, and valvular abnormalities.

However, surgical correction is the definitive treatment for ARCAPA and typically involves reimplantation of the RCA into the aorta or ligation with coronary artery bypass grafting to establish a dual coronary artery supply.^4^ Transferring the right coronary button without tension is challenging to reimplant directly to the anterior wall of the ascending aorta and may need the use of an interposition graft to avoid in-hospital mortality.^5^

In this case, the coexistence of severe bicuspid aortic stenosis and ARCAPA imposed a significant clinical challenge because both conditions can contribute to compromised myocardial perfusion and left ventricle dysfunction. Therefore, our patient underwent urgent surgical intervention that included isolation of RCA from the PA and repair of the PA with a heterogeneous pericardial patch, and mechanical aortic valve replacement with replacement of the aortic root and ascending aorta, along with reimplantation of the anomalous RCA. Short-term and long-term follow-up with annual CT scans and echocardiograms are necessary to assess stenosis or clot in the reimplanted RCA and to identify ventricular dysfunction or myocardial infarction.^5^

## Conclusions

This case highlights the importance of comprehensive preoperative diagnosis of rare congenital anomalies, such as ARCAPA with a bicuspid aortic valve, in adults. Combating this rare but life-threatening condition requires a holistic management plan, including multidisciplinary team meetings with expert panels, prompt surgical interventions, and close postoperative monitoring.

## Conflict of Interest Statement

The authors reported no conflicts of interest.

The *Journal* policy requires editors and reviewers to disclose conflicts of interest and to decline handling or reviewing manuscripts for which they may have a conflict of interest. The editors and reviewers of this article have no conflicts of interest.

## Supplementary Data

Video 1 CT scan showing origin of the right coronary artery originated from the pulmonary artery. Video available at: https://www.jtcvs.org/article/S2666-2507(26)00104-5/fulltext.

Video 2 Aortic root replacement with mechanical aortic valve replacement and ARCAPA correction. Video available at: https://www.jtcvs.org/article/S2666-2507(26)00104-5/fulltext.
