# Supplementary figures and images for: Anomalous origin of the right coronary artery from the pulmonary artery with a bicuspid aortic valve in a 57-year-old patient
Source: JTCVS Tech. 2026 Mar 2;37:102297. doi: 10.1016/j.xjtc.2026.102297 (PMC13261251; doi:10.1016/j.xjtc.2026.102297)

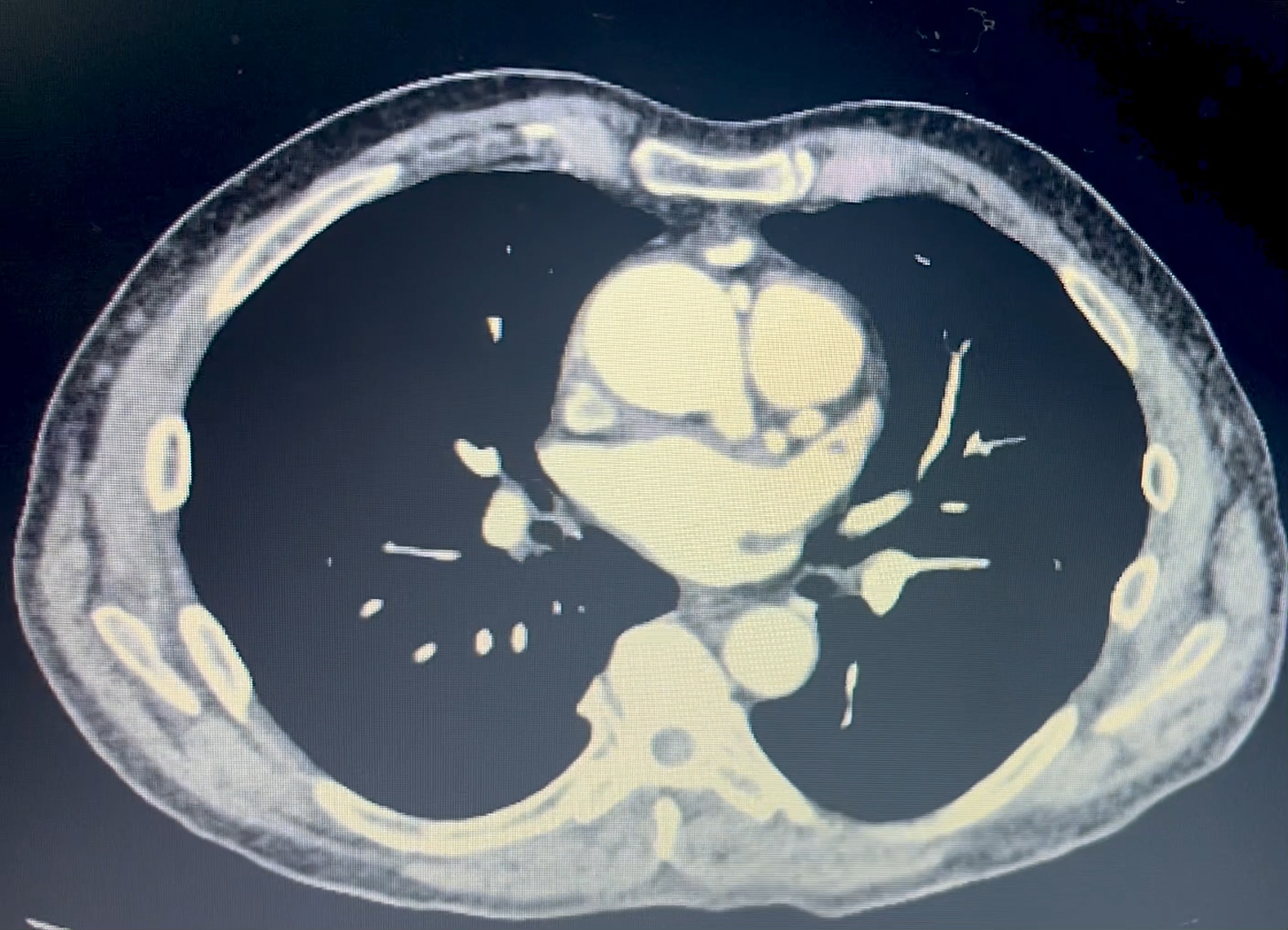

Supplement: Video 1 — CT scan showing origin of the right coronary artery originated from the pulmonary artery. Video available at: https://www.jtcvs.org/article/S2666-2507(26)00104-5/fulltext. [file fx3.jpg]

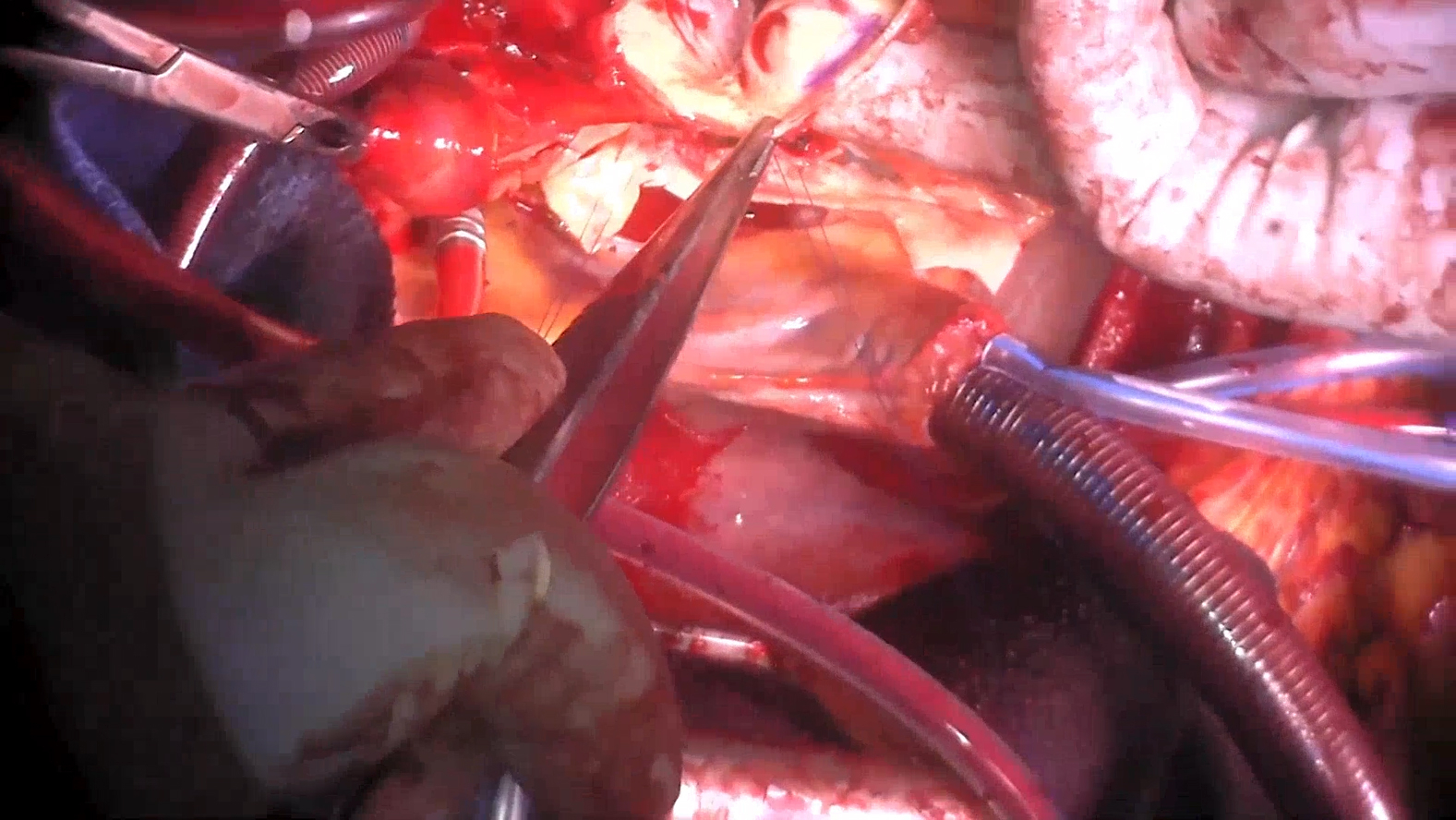

Supplement: Video 2 — Aortic root replacement with mechanical aortic valve replacement and ARCAPA correction. Video available at: https://www.jtcvs.org/article/S2666-2507(26)00104-5/fulltext. [file fx2.jpg]
